# Neuroimmune Crossroads: Pathophysiological Links Between Bipolar Disorder and Inflammatory Bowel Disease

**DOI:** 10.62641/aep.v53i6.2001

**Published:** 2025-12-17

**Authors:** Giuseppe Marano, Francesca Bardi, Emanuela De Chiara, Francesco Maria Lisci, Caterina Brisi, Emanuele Caroppo, Gabriele Sani, Antonio Gasbarrini, Roberto Pola, Eleonora Gaetani, Marianna Mazza

**Affiliations:** ^1^Unit of Psychiatry, Fondazione Policlinico Universitario Agostino Gemelli IRCCS, 00168 Rome, Italy; ^2^Department of Neurosciences, Università Cattolica del Sacro Cuore, 00168 Rome, Italy; ^3^Department of Mental Health, Local Health Authority Roma 2, 00155 Rome, Italy; ^4^Department of Medical and Surgical Sciences, Fondazione Policlinico Universitario Agostino Gemelli IRCCS, Università Cattolica del Sacro Cuore, 00168 Rome, Italy; ^5^Section of Internal Medicine and Thromboembolic Diseases, Department of Internal Medicine, Fondazione Policlinico Universitario Agostino Gemelli IRCCS, Università Cattolica del Sacro Cuore, 00168 Rome, Italy; ^6^Unit of Internal Medicine, Cristo Re Hospital, 00167 Rome, Italy; ^7^Department of Translational Medicine and Surgery, Fondazione Policlinico Universitario Agostino Gemelli IRCCS, Università Cattolica del Sacro Cuore, 00168 Rome, Italy

**Keywords:** bipolar disorder, inflammatory bowel disease, gut-brain axis, immune system, genetic pleiotropy

## Abstract

**Background:**

Bipolar disorder (BD) and inflammatory bowel disease (IBD) frequently co-occur, posing unique treatment challenges and implicating shared inflammatory mechanisms. Although each condition has been extensively studied in isolation, the clinical and pathophysiological interplay between BD and IBD remains poorly characterized.

**Methods:**

We conducted a narrative review of peer-reviewed literature from January 2000 through May 2025, retrieved from PubMed, Web of Science, and PsycINFO. Search terms included “bipolar disorder”, “inflammatory bowel disease”, “comorbidity”, and related inflammatory markers. Titles/abstracts were screened by two reviewers, and eligible studies reporting clinical, epidemiological, or mechanistic data on BD–IBD overlap were included.

**Results:**

Prevalence estimates suggest that BD affects approximately 3–7% of IBD patients, compared with 1–2% in the general population. Comorbid BD–IBD is associated with increased hospitalization rates, more severe gastrointestinal and psychiatric symptoms, and reduced quality of life. Treatment interactions are complex: mood stabilizers and antipsychotics may exacerbate gastrointestinal inflammation, while corticosteroids and biologics can destabilize mood. Mechanistic studies highlight dysregulated cytokine profiles (e.g., elevated Interleukin-6, Tumor Necrosis Factor-alpha I), gut-microbiome alterations, and genetic pleiotropy as convergent pathways.

**Conclusions:**

The intersection of BD and IBD underscores a bidirectional gut–brain neuroimmune axis, with systemic inflammation as a central mediator. Recognizing and managing this comorbidity requires integrated multidisciplinary care. Future research should focus on longitudinal studies and targeted anti-inflammatory interventions to improve outcomes in this high-risk population.

## Introduction

Bipolar disorder (BD) is a chronic mood disorder marked by episodes of depression, mania, hypomania, or mixed states, typically beginning in late adolescence or early adulthood [1, 2]. It affects over 1% of the global population and its prevalence is rising [3]. Given its early onset and lifelong course, BD contributes significantly to disability in young and working-age adults, with major personal, social, and economic impact. Individuals with BD are also at higher risk for systemic comorbidities, including cardiovascular disease, diabetes, respiratory conditions (e.g., Chronic Obstructive Pulmonary Disease), and infections [2, 4, 5]. Emerging evidence suggests a greater prevalence of gastrointestinal disorders, particularly inflammatory bowel disease (IBD), in BD populations [6, 7, 8], supporting a potential link with systemic inflammation beyond the central nervous system.

IBD is a chronic, immune-mediated disorder characterized by persistent inflammation of the gastrointestinal tract. It primarily includes two clinical subtypes: Crohn’s disease (CD) and ulcerative colitis (UC), which differ in their anatomical distribution and histopathological features [9]. The course of IBD is typically relapsing-remitting, marked by alternating flares of active inflammation and periods of clinical remission. Its pathogenesis is multifactorial, involving genetic susceptibility, immune system dysregulation, alterations in the gut microbiota, and environmental triggers [10]. Emerging evidence indicates that the comorbidity of BD may differ between these two major IBD subtypes, suggesting distinct underlying mechanisms [9]. A higher relative risk of BD in UC patients compared with CD has been reported, potentially reflecting differences in mucosal immune activation and microbial ecology [9]. In UC, colonic-restricted inflammation is characterized by broad upregulation of T helper 2 (Th2)-type cytokines (e.g., Interleuchin IL-5, IL-13) alongside elevated IL-6 and C-reactive protein (CRP), cytokine patterns that correlate strongly with depressive symptom burden and cognitive complaints in affected individuals [9]. Conversely, CD exhibits a more heterogeneous cytokine milieu, marked by simultaneous Th1 (InterferonIFN-γ, Tumor Necrosis Factor-alpha) and Th17 (IL-17, IL-23) responses, which aligns with reports of greater anxiety comorbidity and treatment-resistant mood episodes in CD populations [11]. Microbiome analyses further differentiate the subtypes: UC patients often show depletion of butyrate-producing *Firmicutes* (e.g., *Faecalibacterium prausnitzii*), metabolites known to support blood-brain barrier integrity and modulate microglial activation, whereas CD is associated with expansion of adherent-invasive *Escherichia coli* and reduced *Bacteroides* diversity, alterations linked experimentally to altered tryptophan metabolism and serotonin precursor availability. These distinct microbial signatures may therefore differentially influence gut-brain axis signaling and mood regulation [6, 8]. Mechanistically, the subtype-specific cytokine and microbiome landscapes suggest tailored pathways by which systemic inflammation could perturb neuroimmune homeostasis: UC’s Th2-biased environment may preferentially disrupt hippocampal neurogenesis and stress responsivity, whereas CD’s Th1/Th17 predominance might more potently activate microglial inflammasomes, promoting synaptic pruning and cognitive deficits [7, 10].

While IBD primarily affects the gut, it is increasingly recognized as a systemic disease with numerous extraintestinal manifestations, including arthritis, uveitis, and erythema nodosum. Importantly, psychiatric comorbidities, particularly anxiety, depression, and BD, are also commonly reported in IBD patients [12, 13, 14]. In recent years, the relationship between BD and IBD has attracted increasing research interest, but findings have been mixed and often conflicting. Some studies suggest a significantly higher risk of BD in individuals with IBD, supporting the hypothesis of shared pathophysiological mechanisms [15, 16, 17, 18], while other studies have questioned the strength or directionality of this association [19, 20, 21, 22]. A recent meta-analysis by Nikolova *et al*. [23] affirmed that the evidence supporting a consistent association between BD and IBD remains inconclusive.

The frequent co-occurrence of BD and IBD presents notable clinical challenges and suggests a shared inflammatory vulnerability. Psychotropic medications can worsen gastrointestinal symptoms, while IBD treatments like corticosteroids may destabilize mood. This bidirectional risk complicates treatment decisions and increases hospitalization rates. Patients with both conditions often experience greater functional impairment, reduced quality of life, and higher healthcare use than those with either disorder alone. Their overlap provides a valuable model to explore the neuro–immune–endocrine axis. Investigating shared cytokine profiles, gut–brain interactions, and genetic pleiotropy could strengthen the inflammatory hypothesis of mood disorders and guide integrative treatment approaches.

Given the complexity and inconsistency in existing findings, further research is needed. This review summarizes epidemiological evidence on BD–IBD comorbidity, examines treatment implications, explores shared pathophysiology, particularly inflammation and gut–brain neuroimmune signaling, and offers insights for integrated care strategies.

## Methods

A narrative review was conducted to synthesize clinical, epidemiological, and mechanistic studies addressing the comorbidity of BD and IBD. We adhered to PRISMA principles for transparent reporting of literature selection, although formal meta-analysis was not performed. We searched PubMed, Web of Science, and PsycINFO for articles published from January 2000 through May 2025. Search terms combined controlled vocabulary (e.g., MeSH) and keywords for BD (“bipolar disorder”, “manic depression”, “cyclothymia”), IBD (“inflammatory bowel disease”, “Crohn’s disease”, “ulcerative colitis”), and comorbidity (“comorbid”, “overlap”, “inflammation”). Boolean operators (AND/OR) were used to maximize sensitivity. Reference lists of key reviews and retrieved full-texts were hand-searched to identify additional relevant studies. Studies were eligible if they were peer-reviewed, reported clinical, epidemiological, or pathophysiological data on BD–IBD comorbidity, included adult human subjects or relevant mechanistic animal models. We excluded case reports or series with fewer than five participants and publications focusing exclusively on psychiatric or gastrointestinal conditions other than BD or IBD. Titles and abstracts were screened independently by two authors (MM and GM); disagreements were resolved by consensus. Although formal scoring tools were not applied, we evaluated study rigor by noting sample size, diagnostic criteria validity, and statistical adjustment for confounders.

### Shared Clinical Features Between BD and IBD

From a clinical standpoint, BD and IBD exhibit several overlapping characteristics, suggesting potential shared pathophysiological mechanisms. Both conditions follow a relapsing-remitting course, marked by alternating periods of symptomatic exacerbation and remission [24, 25]. In both BD and IBD, episodes of relative stability are frequently interrupted by acute flare-ups, each contributing to cumulative disease burden and functional impairment [3, 26].

Environmental stressors, particularly psychological stress, are known to play a significant role in triggering exacerbations in both disorders. Stress has been consistently implicated in the onset, severity, and recurrence of symptoms in both BD and IBD, underscoring a shared vulnerability to external factors [27, 28]. This stress sensitivity further complicates disease management, especially during critical transitional periods such as diagnosis, major life events, or treatment changes.

In terms of symptomatology, IBD extends beyond the gastrointestinal tract, encompassing a range of psychiatric comorbidities, including anxiety, depression, and BD [29, 30]. A recent meta-analysis reported pooled prevalence rates of 32.1% for anxiety symptoms and 25.2% for depressive symptoms among individuals with IBD, highlighting the considerable psychiatric burden in this population [11]. These mood symptoms often precede or accompany IBD flare-ups, suggesting that psychological distress may not merely result from IBD symptoms but may actively contribute to disease exacerbation [31].

The temporal proximity of psychiatric symptoms to IBD diagnosis is another clinically relevant observation. Several studies indicate that affective symptoms, including those resembling BD, are especially prevalent around the time of IBD diagnosis. For instance, Bisgaard *et al*. [21] demonstrated that patients withCD had a significantly higher likelihood of psychiatric consultations in the years following diagnosis. This may partly reflect the shared age of onset, as both BD and IBD typically emerge in young adulthood, with a common peak between ages 15 to 30 for both CD and UC [32]. Moreover, the psychological impact of receiving a chronic illness diagnosis may act as a trigger for mood disturbances, further reinforcing the bidirectional link between psychiatric symptoms and gastrointestinal inflammation [32, 33, 34].

Beyond mood symptoms, both BD and IBD are associated with neurovegetative and cognitive disturbances. Cognitive dysfunction-including difficulties with attention, memory, and executive function-is a well-documented feature of BD, even during euthymic phases [35, 36]. Similarly, IBD patients often report cognitive complaints such as mental fog and concentration difficulties [37, 38]. While these cognitive issues in IBD are frequently attributed to systemic inflammation, fatigue, or nutritional deficiencies, few studies have examined them through objective neuropsychological testing or neuroimaging.

Sleep disturbances also represent a key point of overlap. In BD, disrupted sleep is both a core symptom and a predictor of mood episode recurrence [39]. In IBD, poor sleep quality is common, often related to nocturnal symptoms, pain, or anxiety. Importantly, sleep disturbance in IBD can contribute to immune system dysregulation, worsening gastrointestinal inflammation and potentially creating a self-perpetuating cycle of disease activity [40]. The convergence of cognitive and sleep-related symptoms in both disorders reinforces the likelihood of shared underlying mechanisms, including inflammation, circadian dysregulation, and gut-brain axis disruption.

Although BD and IBD share an array of overlapping symptoms, their concurrent management raises unique challenges. The very manifestations that link these disorders at the clinical level also create points of therapeutic tension: interventions targeting one symptom domain may inadvertently exacerbate another. For example, efforts to normalize mood through corticosteroids can worsen gastrointestinal inflammation, while antipsychotic-induced metabolic changes may aggravate IBD activity [41, 42]. It is therefore critical to recognize how symptom commonality underpins, and indeed complicates, every subsequent decision about pharmacologic and non-pharmacologic management in comorbid patients.

From a treatment perspective, the co-occurrence of BD and IBD presents both therapeutic challenges and opportunities. Several psychotropic medications, particularly mood stabilizers, have gastrointestinal side effects that may complicate IBD management. Lithium, a first-line treatment for BD, is known to cause nausea, diarrhea, and, in rare cases, may exacerbate inflammatory conditions of the gastrointestinal tract [43, 44, 45]. These side effects raise concerns regarding its safety profile in patients with comorbid IBD, where maintaining mucosal healing is a primary treatment goal [46].

Conversely, corticosteroids, frequently used to manage acute IBD flares, are well known for their psychiatric side effects, including mood swings, insomnia, and even steroid-induced mania or depression [47, 48]. These effects are particularly relevant for individuals with underlying mood disorders, including BD, where corticosteroid treatment may precipitate affective episodes [49]. Careful psychiatric monitoring and risk-benefit evaluation are essential when using steroids in this population.

In addition, gastrointestinal side effects of antidepressants (e.g., effects on bowel motility) may require clinicians to tailor antidepressant therapy according to individual bowel habits, especially in patients with comorbid irritable bowel symptoms [26]. Encouragingly, the effective treatment of anxiety and depression has been shown to improve IBD outcomes and enhance quality of life, further highlighting the interconnected nature of psychiatric and gastrointestinal health [17, 26, 50, 51, 52]. Table 1 (Ref. [3, 5, 9, 13, 34, 36, 53, 54, 55, 56, 57, 58, 59, 60, 61, 62]) shows shared Clinical Features Between BD and IBD: prevalence estimates are approximate and derived from multiple clinical studies. In comorbid patients, these overlapping features often interact synergistically, leading to greater overall disease burden and necessitating integrated management approaches.

**Table 1.** **Shared clinical features between bipolar disorder and inflammatory bowel disease**.

Feature	Bipolar disorder (BD)	Inflammatory bowel disease (IBD)	Impact in comorbidity
Fatigue [53, 54]	Persistent low energy during depressive and inter-episode phases (up to 60% of patients)	Chronic fatigue reported in 40–70% of patients, correlating with disease activity	Exacerbated functional impairment; increased risk of depressive relapse and reduced adherence to IBD therapy
Sleep disturbance [55, 56]	Insomnia in mania/hypomania; hypersomnia in depression (50–80% prevalence)	Sleep fragmentation and poor sleep quality reported by ~50% of patients, especially during flares	Worsened mood regulation and gastrointestinal symptom control
Appetite changes [57, 58]	Hyperphagia in mania; anorexia in depression	Weight loss, decreased appetite during active disease in up to 70% of cases	Nutritional deficits contribute to mood instability and medication tolerability
Abdominal pain [59, 60]	Somatic pain complaints, including abdominal discomfort, in up to 30% of patients	Cramping and abdominal pain in >80% of patients during active flares	Heightened pain sensitivity; challenges in distinguishing psychiatric vs. organic pain sources
Mood dysregulation [61, 62]	Core feature: swings between mania, hypomania, and depression	Secondary mood symptoms (anxiety/depression) in 30–50% of patients	Bidirectional exacerbation: stress exacerbates flares, flares worsen mood
Cognitive impairment [3, 34, 36]	Deficits in attention, memory, and executive function in ~40% of patients	“Brain fog” reported by ~20–40%, particularly during active inflammation	Impairs self-management of both conditions; increases healthcare utilization
Inflammatory markers [5, 9, 13]	Elevated cytokines (e.g., IL-6, TNF-α) in blood and CSF in >50% of episodes	Elevated systemic cytokines and CRP in >70% of active disease	Shared inflammatory milieu suggests common pathophysiological targets

Abbreviations: IL-6, Interleukin-6; TNF-α, Tumor necrosis factor-alpha; CSF, cerebrospinal fluid; CRP, C-reactive protein.

## Inflammatory Pathways in BD and IBD

An expanding body of scientific literature supports the notion that systemic inflammation acts as a shared pathophysiological mechanism underlying both BD and IBD. Although these conditions have traditionally been viewed as distinct in etiology and clinical presentation, emerging data suggest that chronic, low-grade inflammation may play a central role in the development and progression of both. This inflammatory hypothesis offers a compelling framework for understanding their frequent co-occurrence and overlapping symptomatology.

### Inflammatory Activity in BD

Over the past decade, research into BD has increasingly focused on identifying immunological alterations that may contribute to the disorder’s pathogenesis, course, and treatment response. A particular emphasis has been placed on characterizing peripheral cytokine profiles across different mood states, with the aim of identifying biomarkers for diagnosis, prognosis, or treatment monitoring [63].

Several studies have demonstrated that individuals with BD exhibit a mood-dependent inflammatory profile [64, 65, 66, 67]. Even during euthymia, patients often present with a mild pro-inflammatory state, characterized by elevated soluble tumor necrosis factor receptor 1 (sTNFR1) and, in some cases, increased plasma concentrations of C-X-C motif chemokine ligand 10 (CXCL10, also known as interferon gamma-induced protein 10 or IP-10). This pattern suggests a possible shift toward T helper 1 (Th1)-mediated immune activation [63, 64].

Inflammatory alterations become more pronounced during acute mood episodes. In mania, several studies have reported elevated levels of IL-6, TNF-α, sTNFR1, IL-1 receptor antagonist (IL-1ra), IL-4, CXCL10, and CXCL11, suggesting activation of both Th1 and Th2 pathways [65, 66, 67, 68]. However, some inconsistencies remain. For example, Kim *et al*. [69] observed increased IL-6 and TNF-α but decreased IL-4 levels during mania, highlighting inter-study variability possibly due to sample heterogeneity, medication status, or methodological differences.

During the depressive phase, elevated levels of sTNFR1 and CXCL10 have also been documented, although fewer studies have focused on inflammatory markers in bipolar depression compared to mania or euthymia [65, 70]. Importantly, there are findings suggesting that pharmacological treatment may modulate inflammatory activity, with reductions in IL-6 levels following mood stabilization [68, 69, 70]. Collectively, these observations support the idea that BD is characterized by cytokine imbalance, which fluctuates according to clinical state and treatment status, and may contribute to both core mood symptoms and somatic comorbidities.

### Inflammatory Signaling in IBD

Similarly, inflammation plays a central role in IBD pathogenesis, with both CD and UC exhibiting profound dysregulation of innate and adaptive immune responses. Genome-wide association studies (GWAS) have identified numerous risk loci that encode key regulators of cytokine production and immune signaling, such as nucleotide-binding oligomerization domain-containing protein 2 (NOD2), Interleukin-23 receptor (IL23R), and signal transducer and activator of transcription 3 (STAT3), emphasizing the genetic underpinnings of IBD-related inflammation [70, 71].

The pro-inflammatory cytokine TNF-α is a pivotal driver of intestinal inflammation in IBD and serves as a major therapeutic target in biologic treatments [59]. Other cytokines involved include IL-6, IL-12, IL-23, and IL-21, while anti-inflammatory mediators such as IL-10 and TGF-β are often impaired [70]. The resulting cytokine imbalance not only drives intestinal tissue damage but also contributes to systemic manifestations, including arthralgia, fatigue, and neuropsychiatric symptoms, reflecting the disease’s extraintestinal burden [70].

Beyond the broad characterization of inflammatory phenotypes, several well-defined signaling cascades have been implicated in both BD and IBD, suggesting convergent molecular mechanisms: nuclear Factor kappa-light-chain-enhancer of activated B cells pathway (NF-κB pathway), Janus kinase (JAK)–signal transducer and activator of transcription (STAT) cascade (JAK–STAT pathway), and NLRP3 inflammasome (NOD-, LRR-, and pyrin domain-containing protein 3 inflammasome).

In response to stressors or microbial products, toll-like receptors (TLRs) and cytokine receptors activate the IκB kinase complex (IKK complex), leading to degradation of IκB and nuclear translocation of NF-κB transcription factors. In the central nervous system, NF-κB drives microglial release of IL-6, TNF-α, and IL-1β, which can alter synaptic plasticity and contribute to mood dysregulation. In the gut, epithelial and immune cell NF-κB signaling upregulates chemokines (e.g., C-C motif chemokine ligand 2 or CCL2) and antimicrobial peptides, perpetuating mucosal inflammation and barrier dysfunction. Dysregulated NF-κB activity has been detected in postmortem BD brain tissue and inflamed IBD mucosa, reinforcing its role as a shared node of neuro-immune crosstalk [70, 71, 72].

Many pro-inflammatory cytokines (e.g., IL-6, IFN-γ) signal via Janus kinases (JAK1/2) and subsequent phosphorylation of STAT transcription factors. In BD, peripheral blood mononuclear cells exhibit heightened STAT3 phosphorylation in response to IL-6 stimulation, which correlates with acute mood episodes. In IBD, JAK–STAT signaling, particularly via STAT1 and STAT3, regulates T helper cell differentiation (Th1/Th17) and epithelial apoptosis. The clinical efficacy of JAK inhibitors (e.g., tofacitinib) in UC further underscores the pathway’s centrality, and emerging pilot trials of JAK blockade in treatment-resistant BD hint at cross-disorder therapeutic potential [71, 73].

The NLRP3 complex senses cellular “danger” signals (e.g., adenosine triphosphate-ATP, crystalline structures), recruiting apoptosis-associated speck-like protein (ASC) and pro-caspase-1 to catalyze IL-1β and IL-18 maturation. In BD patients, elevated serum IL-1β and increased monocyte NLRP3 expression have been observed during manic and depressive phases, suggesting systemic inflammasome priming. In IBD, NLRP3 activation within macrophages and intestinal epithelial cells contributes to mucosal damage and dysbiosis; blockade of caspase-1 in murine colitis models ameliorates disease severity. Given its dual involvement, the NLRP3 inflammasome represents a promising target for interventions aimed at dampening both neuroinflammation and gut pathology [67, 73].

### Converging Mechanisms and Clinical Implications

Although BD and IBD differ in primary organ involvement-the brain and the gut, respectively-both are marked by immune-inflammatory activation that contributes to the manifestation and severity of clinical symptoms [74, 75]. In IBD, inflammation is initially localized to gastrointestinal mucosa but may secondarily affect distant systems via circulating cytokines and immune cells. In contrast, in BD, inflammation appears to be more systemic and neurocentric, involving the central nervous system (CNS) through microglial activation, blood–brain barrier disruption, and altered neurotransmitter signaling [76].

Despite these differences in topography, both conditions involve shared inflammatory mediators, including TNF-α and IL-6, and likely converge at the level of the neuro–immune–endocrine axis. This axis integrates signals from the brain, gut, and immune system, offering a mechanistic link between emotional regulation, intestinal health, and systemic inflammation [52].

These insights support the growing view that BD and IBD may represent two clinical expressions of a shared inflammatory vulnerability, modulated by genetics, environment, and microbiota (Table 2).

**Table 2.** **Cytokine function and presence in bipolar disorder (BD) and inflammatory bowel disease (IBD)**.

Cytokine	Function	Intensity	Presence (BD/IBD/Shared)
TNF-α	▲ Pro-inflammatory	••• Strong	BD, IBD, Shared
IL-1β	▲ Pro-inflammatory	•• Moderate	BD, IBD
IL-2	▲ Pro-inflammatory	• Mild	BD
IL-4	▼ Anti-inflammatory	• Mild	IBD
IL-6	▲ Pro-inflammatory	•• Moderate	BD, IBD, Shared
IL-8	▲ Pro-inflammatory	•• Moderate	BD, Shared
IL-10	▼ Anti-inflammatory	• Mild	BD, IBD, Shared
IL-12	▲ Pro-inflammatory	•• Moderate	BD
IL-17	▲ Pro-inflammatory	•• Moderate	IBD
IL-18	▲ Pro-inflammatory	•• Moderate	BD
IFN-γ	▲ Pro-inflammatory	••• Strong	BD, Shared
CRP	▲ Pro-inflammatory	••• Strong	BD, IBD, Shared

This table summarizes the involvement of key cytokines in bipolar disorder (BD), inflammatory bowel disease (IBD), or both. Each cytokine is characterized by function and intensity. Function: ▲ Pro-inflammatory: Promotes inflammatory response; ▼ Anti-inflammatory: Regulates or suppresses inflammation. Intensity: • Mild: Low elevation or limited impact; •• Moderate: Moderate expression or impact; ••• Strong: Robust elevation and strong pathogenic role. Presence: Indicates whether the cytokine has been implicated in BD, IBD, or in both conditions (“Shared”) based on current evidence.

While the role of gut microbial dysbiosis in the pathogenesis of IBD is well established-characterized by chronic inflammation, disrupted mucosal integrity, and increased intestinal permeability [77, 78], growing evidence suggests that the gut microbiota also exerts a profound influence on emotional, cognitive, and behavioral functions [75, 79, 80, 81]

At the center of this interaction lies the microbiota–gut–brain axis, a bidirectional communication network that links the gastrointestinal tract with the CNS. This system is mediated by a complex interplay of neuroendocrine, immune, metabolic, and neural signaling pathways [75, 77]. In mood disorders such as BD, stress-related activation of the hypothalamic–pituitary–adrenalaxis results in the release of corticotropin-releasing factor (CRF), adrenocorticotropic hormone, and glucocorticoids. This cascade increases intestinal permeability, promotes mast cell degranulation, and stimulates the production of pro-inflammatory cytokines [80, 81]. The breakdown of intestinal barrier integrity facilitates the translocation of microbial products into the systemic circulation, contributing to peripheral immune activation and low-grade inflammation, processes increasingly recognized in the pathophysiology of mood disorders including BD.

In this context, microbial dysbiosis may serve as a common driver of chronic inflammation in both BD and IBD. Notably, interventions that target the gut microbiota, such as dietary modifications, prebiotics, probiotics, and fecal microbiota transplantation, have shown potential in modulating affective symptoms, thereby opening the door to microbiota-targeted therapies in psychiatric disorders [82, 83].

Recent studies have highlighted the potential role of fungal dysbiosis in the pathophysiology of both IBD and BD [84, 85, 86, 87]. In particular, alterations in *Candida albicans* and *Saccharomyces cerevisiae* populations have been reported across both conditions. In IBD, increased levels of *Candida albicans* are commonly observed, reflecting fungal overgrowth and mucosal immune activation [84]. Similarly, *Saccharomyces cerevisiae* has been found in greater abundance in UC, and antibodies directed against it-anti-Saccharomyces cerevisiae antibodies (ASCAs)-have been widely investigated as serological markers for CD [85, 86, 87].

Strikingly, similar findings have emerged in mood disorders. Elevated levels of both *Candida albicans* and *Saccharomyces cerevisiae* have been reported in individuals with BD and major depressive disorder, mirroring microbial trends seen in IBD [88]. Severance *et al*. [89] found that ASCA levels were significantly higher in BD patients compared to healthy controls, independent of pharmacological treatment. Originally intended as a diagnostic marker for IBD, the presence of ASCAs in BD patients suggests that fungal antigens and associated immune responses may represent transdiagnostic biomarkers of gut-derived immune dysregulation. These findings support the hypothesis that shared inflammatory processes involving microbial-host interactions may underlie both psychiatric and gastrointestinal disorders [22].

In addition to fungal components, bacterial dysbiosis is a critical feature in both disease spectrums. Individuals with BD have been shown to exhibit a decreased abundance of *Faecalibacterium*, genus known for its anti-inflammatory properties, and increased levels of *Actinobacteria*, compared to healthy controls [90]. These microbial alterations parallel those observed in IBD and are associated with heightened systemic inflammation and altered neuroimmune signaling. Given microbiota’s role in modulating the immune system, neurotransmitter production, and gut permeability, such dysbiosis could influence both gastrointestinal and psychiatric symptoms.

The gut microbiota contributes directly to neurochemical signaling by producing and modulating a variety of neurotransmitters, including γ-aminobutyric acid (GABA), serotonin, dopamine, and norepinephrine [91, 92, 93]. Specific microorganisms such as *Escherichia spp*. can synthesize norepinephrine and serotonin, while *Candida*, *Streptococcus*, and *Enterococcus spp*. are known to produce serotonin [94, 95]. Remarkably, over 90% of the body’s serotonin is produced in the gastrointestinal tract, highlighting the central role of the enteric nervous system and the gut microbiota in regulating both gut function and mood [96].

The interaction between the gut and CNS is bidirectional. While the microbiota influences brain function, the CNS in turn modulates gastrointestinal physiology through autonomic, sensory, and hormonal pathways. For example, serotonin, which enhances gastrointestinal motility, is elevated in both the mucosa and peripheral circulation of IBD patients, particularly those with CD [96, 97, 98]. Dysregulation of serotonin homeostasis may thus play a role in both abnormal gut motility and mood instability, further bridging the two conditions.

Taken together, these findings support the existence of a shared pathophysiological framework linking BD and IBD through the gut–immune–brain axis. This includes bacterial and fungal dysbiosis, immune activation (e.g., elevated ASCAs), and altered neurotransmitter signaling, all of which may contribute to systemic inflammation and neurobehavioral changes (Table 3, Ref. [14, 22, 37, 75, 79, 81, 83, 84, 85, 86, 88, 89, 90, 91, 94]). These insights emphasize the transdiagnostic relevance of the gut-brain axis and open new avenues for integrative therapeutic strategies targeting the microbiome to simultaneously address gastrointestinal and psychiatric symptoms.

**Table 3.** **Microbial and immune findings in inflammatory bowel disease (IBD) and bipolar disorder (BD)**.

Component	Findings in IBD	Findings in BD	References	Research type
Candida albicans	Increased abundance; associated with mucosal immune activation	Increased levels reported; linked to immune activation	Sokol *et al*., 2017 [84]; McGuinness *et al*., 2024 [88]	Cross-sectional mycobiome profiling [84]; cross-sectional serological profiling [88]
Saccharomyces cerevisiae	Elevated in UC; target of ASCA antibodies	Elevated levels in BD and MDD; similar trends as in IBD	Chiaro *et al*., 2017 [85]; McGuinness *et al*., 2024 [88]	Case–control serological study [85]; cross-sectional profiling [88]
ASCAs	Commonly elevated in CD; used as a serological marker	Elevated levels observed; potential marker of gut-derived immune response	Peeters *et al*., 2001 [86]; Severance *et al*., 2014 [89]	Case–control serological study [86, 89]
Faecalibacterium spp.	Reduced levels; associated with loss of anti-inflammatory activity	Reduced levels; associated with systemic inflammation	Huang *et al*., 2019 [90]	Cross-sectional microbiome sequencing study [90]
Actinobacteria	Not typically reported in IBD context	Increased levels; associated with microbial dysbiosis	Huang *et al*., 2019 [90]	Cross-sectional microbiome sequencing study [90]
Escherichia spp.	Produces serotonin and norepinephrine; may affect gut–brain signaling	Produces neuroactive compounds; implicated in neurotransmitter balance	Cryan and Dinan, 2012 [94]	Narrative review [94]
Streptococcus spp.	Produces serotonin; may affect gut–brain signaling	Produces serotonin; potential role in mood regulation	Cryan and Dinan, 2012 [94]	Narrative review [94]
Enterococcus spp.	Produces serotonin; may affect gut–brain signaling	Produces serotonin; potential role in mood regulation	Cryan and Dinan, 2012 [94]	Narrative review [94]
Bacteroides spp.	Altered abundance; some species associated with inflammation	Altered ratios observed; may influence mood via SCFA production	Kostic *et al*., 2014 [75]; Liu *et al*., 2019 [83]	Cross-sectional microbiome sequencing studies [75, 83]
Lactobacillus spp.	Often decreased; contributes to mucosal health and anti-inflammatory effects	Reduced levels; associated with impaired gut–brain signaling	Barrett *et al*., 2012 [91]; Liu *et al*., 2019 [83]	Experimental culture study [91]; cross-sectional profiling [83]
Ruminococcus spp.	Decreased levels; linked to reduced production of short-chain fatty acids (SCFAs)	Reduced abundance; linked to cognitive and emotional dysfunction	Huang *et al*., 2019 [90]; Zielinski *et al*., 2019 [37]	Cross-sectional study [90]; narrative review [37]
Clostridium cluster IV/XIVa	Reduced diversity; associated with impaired gut barrier function	Decreased abundance; associated with inflammation and mood instability	Osadchiy *et al*., 2019 [79]	Cross-sectional clinical microbiome analysis [79]
Prevotella spp.	Variable findings; increased in some IBD phenotypes	Altered abundance; possibly modulates host immune responses	Marano *et al*., 2025 [14]	Narrative review [14]
IL-6	Elevated levels; associated with active inflammation	Elevated levels in mood episodes; contributes to systemic inflammation	Wang *et al*., 2022 [22]	Cross-sectional biomarker study [22]
TNF-α	Consistently elevated; key cytokine in IBD pathogenesis	Elevated levels in BD; correlated with affective symptom severity	Wang *et al*., 2022 [22]; McGuinness *et al*., 2024 [88]	Cross-sectional biomarker studies [22, 88]
Zonulin	Elevated; marker of increased intestinal permeability	Increased levels; associated with disrupted gut barrier and inflammation	Hill *et al*., 2013 [81]	Case–control biomarker study [81]

Abbreviations: ASCAs, Anti-Saccharomyces cerevisiae antibodies; BD, Bipolar Disorder; CD, Crohn’s disease; IBD, inflammatory bowel disease; MDD, Major Depressive Disorder; UC, ulcerative colitis.

## Genetic Correlations and Immune-Related Pleiotropy in BD and IBD

Genetic investigations offer a powerful lens for disentangling the BD–IBD relationship by identifying shared heritable risk factors that point to common biological pathways. By uncovering pleiotropic loci and mapping their functional consequences in immune and neural cell types, genetic research not only illuminates mechanistic overlap but also highlights candidate targets for cross-disorder therapeutics. The role of genetics in both BD and IBD has been extensively studied in isolation [99, 100]. However, the investigation of shared genetic architecture between these two complex conditions remains relatively limited.

One of the earliest studies to advocate for an integrative genetic approach across traits was conducted by Zhu *et al*. [101], who introduced the Summary-data-based Mendelian Randomization method. Although not focused specifically on BD or IBD, this methodological framework demonstrated how combining GWAS data with expression quantitative trait locus analyses can help prioritize genes whose expression may influence multiple traits due to pleiotropy. This laid the groundwork for later studies exploring shared genetic determinants between psychiatric and immune-mediated disorders.

Building upon this conceptual foundation, recent investigations have adopted cross-trait meta-analyses and genetic correlation analyses to assess potential overlap between BD and IBD [18, 22, 102, 103, 104]. Multiple independent genetic variants have been demonstrated to confer risk for both BD and IBD, indicating pleiotropic effects. Among these, loci on chromosome 1p13.2 and within the major histocompatibility complex (MHC) region were prominent. These genomic regions are well-known for their roles in immune regulation, and have been previously associated with neuropsychiatric, autoimmune, and inflammatory disorders [104, 105]. The identification of such loci underscores the converging roles of immunity and neuroinflammation in both BD and IBD.

Wang *et al*. [18] recently identified five novel pleiotropic genes shared between BD and IBD: *Zinc Finger DHHC-Type Palmitoyltransferase 2* (ZDHHC2), *Secernin-1* (SCRN1), *Inositol Polyphosphate-4-Phosphatase Type II B* (INPP4B), *Chromosome 1 Open Reading Frame 123* (C1orf123), and *Bromodomain-Containing Protein 3* (BRD3). These genes are implicated in a range of biological processes, notably: ZDHHC2: involved in palmitoylation of membrane proteins, with emerging roles in synaptic plasticity and immune signaling [18, 105]; SCRN1: linked to endosomal trafficking and innate immunity [106]; INPP4B: a phosphatase involved in PI3K signaling and inflammatory modulation [107]; C1orf123: a lesser-known gene with emerging links to T-cell signaling [108]; BRD3: a bromodomain-containing protein involved in epigenetic regulation of gene expression and implicated in neurodevelopment [109].

In recent genome-wide association studies, variants in ZDHHC2 and SCRN1 have emerged as loci of interest for both BD and IBD [18, 105, 106]. Although these findings are based on indirect association, they offer intriguing mechanistic hypotheses that merit cautious interpretation. ZDHHC2 catalyzes S-palmitoylation of cysteine residues on client proteins, a reversible lipid modification that regulates protein trafficking and membrane localization [18, 105]. In immune cells, ZDHHC2-mediated palmitoylation of TLRsand adaptor proteins (e.g., myeloid differentiation primary response 88 or MyD88) enhances receptor clustering in lipid rafts and potentiates downstream NF-κB signaling. Dysregulation of this process could therefore amplify systemic inflammatory responses, providing a plausible link to IBD pathogenesis. In parallel, aberrant palmitoylation of neuronal ion channels and synaptic scaffolding proteins could disrupt synaptic plasticity and mood regulation in BD. While direct functional studies in patient-derived cells are lacking, the convergence of GWAS signals with known ZDHHC2 substrates supports a potential shared pathophysiological role in BD–IBD comorbidity.

SCRN1 modulates endosomal trafficking and antigen presentation by influencing the maturation of MHC class II–containing compartments. In gut-associated lymphoid tissue, altered SCRN1 expression may shift the balance between tolerance and immunogenicity to luminal antigens, thereby contributing to mucosal inflammation characteristic of IBD. Within the central nervous system, dysregulated endosomal handling in microglia could affect presentation of neoantigens or clearance of debris, promoting neuroimmune perturbations implicated in BD [103]. Although current evidence rests on expression quantitative trait loci (eQTL) correlations and animal model data, these mechanistic insights underscore SCRN1 as a hypothetical mediator of systemic inflammation across both disorders [18, 105, 106, 109].

These findings emphasize that the genetic intersection between BD and IBD lies primarily within immune-related and neurodevelopmental pathways, further strengthening the hypothesis of shared inflammatory and neurobiological substrates. Despite these overlaps, Mendelian randomization analyses conducted by Wang *et al*. [18] did not support a direct causal relationship between BD and IBD. Instead, the authors proposed that shared risk variants may act on common intermediate phenotypes, such as immune dysregulation, microglial activation, or gut-brain axis disturbances, rather than indicating that one condition causally contributes to the development of the other. This nuance is critical for interpreting genetic correlation findings, as pleiotropy can reflect biological intersection rather than causation.

Overall, these results highlight the importance of integrating multi-omics approaches, including genomics, transcriptomics, proteomics, and epigenetics, to unravel the shared and disease-specific molecular pathways underlying BD and IBD. Moving forward, such integrative studies may uncover new biomarkers and therapeutic targets with relevance across psychiatric and immune-mediated conditions. Future work employing CRISPR-mediated allele editing in immune and neuronal cell types, coupled with palmitoylation and antigen-presentation assays, will be critical to validate the causal contribution of ZDHHC2 and SCRN1 to BD–IBD pathophysiology.

## Sources of Inconsistent Findings in BD–IBD Research

Despite growing evidence linking BD and IBD, findings have often been contradictory, reflecting a constellation of methodological and biological factors. Bidirectional associations complicate causal inference: mood episodes can precipitate gastrointestinal flares via stress-mediated immune activation, while IBD exacerbations may trigger mood destabilization through systemic inflammation and gut–brain axis signaling [6, 7]. Heterogeneity in cohort demographics, including age at enrollment, ethnicity, and socioeconomic status, affects baseline risk profiles for both conditions and may bias prevalence estimates [15, 16]. Besides, variations in diagnostic criteria and the use of administrative claims data versus structured clinical assessments introduce classification inconsistencies. Also, medication use differs substantially across studies: lithium and antipsychotics can influence gut permeability and microbiota composition, while corticosteroids and biologics modulate central cytokine levels, confounding associations between disease activity and inflammatory markers [43, 44]. Finally, unmeasured lifestyle confounders, such as smoking, diet, and physical activity, vary across populations and can independently impact both mood and gut inflammation. By accounting for these factors, through stratified analyses, standardized diagnostic protocols, and comprehensive covariate adjustment, efforts in ongoing studies can move beyond simple enumeration of associations toward a more nuanced understanding of BD–IBD comorbidity [16, 17].

## Results From Existing Literature

### Clinical Overlap and Therapeutic Challenges

The emerging interplay between BD and IBD provides a valuable framework for rethinking the nosology of mood and immune-mediated disorders through a cross-diagnostic, integrative lens. This narrative review highlights converging clinical, immunological, microbial, and genetic features that suggest potential shared pathophysiological mechanisms [2, 4, 5]. Despite a growing body of literature supporting this association, several important limitations and unanswered questions continue to constrain the interpretability and clinical applicability of current findings.

Clinically, BD and IBD share key characteristics, including a relapsing–remitting course, sensitivity to environmental stressors, and overlapping psychiatric symptoms. Notably, affective symptoms often co-occur with IBD, with some evidence suggesting they may precede or exacerbate disease flare-ups. Additional shared symptoms (cognitive dysfunction, fatigue, and sleep disturbances) are frequently reported in both disorders [12, 14]. The clinical management of comorbidity between BD and IBD poses challenges, especially given potential pharmacological interactions. There is growing evidence that effective treatment of psychiatric symptoms in IBD may enhance overall disease outcomes, reinforcing the need for integrated care.

### Pathophysiological Mechanisms

#### Systemic Inflammation

From a pathophysiological perspective, systemic inflammation emerges as a core shared feature. In BD, peripheral cytokine levels fluctuate with mood state, with elevations in both pro- and anti-inflammatory markers across manic, depressive, and euthymic phases. Parallel inflammatory responses (including increased levels of TNF-α, IL-6, and other cytokines) are central to gut inflammation in IBD [6, 9]. The immune activation and cytokine imbalance observed in both disorders are likely to contribute to clinical symptomatology and disease progression.

#### Gut–Microbiota Axis

The role of the gut microbiota in mediating gut-brain communication offers another layer of connection. Both bacterial and fungal dysbiosis are observed in BD and IBD. Disruptions in gut-brain signaling are further mediated by increased intestinal permeability, neurochemical imbalances, and impaired enteric immune regulation, all closely tied to microbial alterations. These findings support a model in which gut microbial dysbiosis may be a transdiagnostic feature of both disorders [7, 10].

#### Genetic Insights

At the genetic level, recent studies have revealed shared risk loci and pleiotropic genes between BD and IBD, particularly those involved in immune signaling and neurodevelopment. However, Mendelian randomization analyses have not established a direct causal relationship, suggesting these shared genetic factors may act through common intermediate phenotypes, such as immune dysregulation or neuroinflammation, rather than indicating a causal link between the disorders themselves [18].

**Fig. 1.**
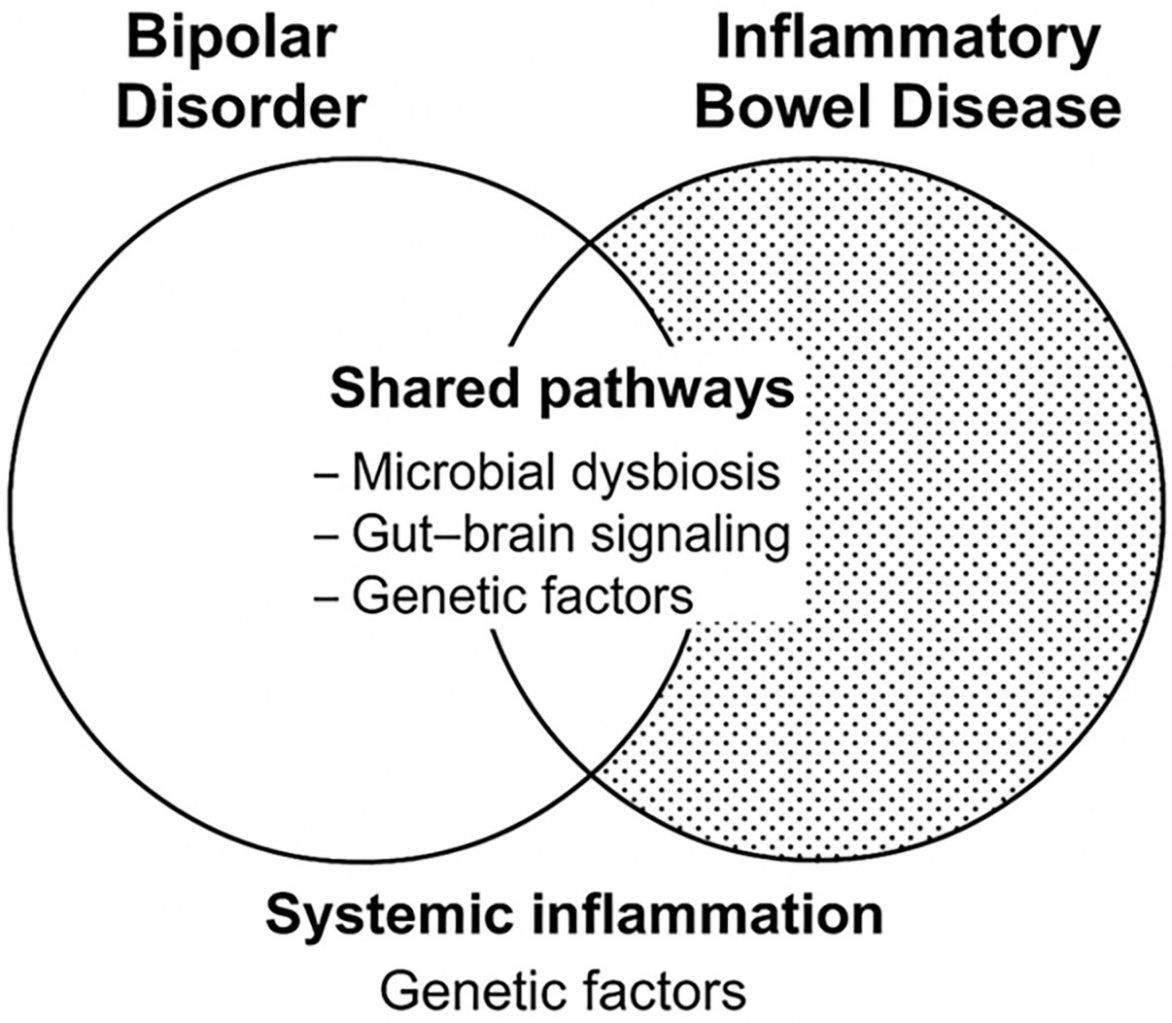
**Hierarchical model of BD–IBD pathophysiology**. This figure illustrates a hierarchical cascade linking genetic and environmental factors to the pathogenesis of bipolar disorder (BD) and inflammatory bowel disease (IBD). Genetic predisposition and environmental triggers (e.g., stress, diet, infections) converge to activate systemic inflammation. From this point, two interconnected pathways emerge: the neuro–immune axis, where inflammatory mediators influence brain function and contribute to BD; the gut–brain axis, involving bidirectional interactions between intestinal inflammation and neural signaling, promoting IBD.

All these findings suggest a nuanced and multifactorial relationship between BD and IBD. While evidence from multiple domains (epidemiological, clinical, immunological, microbiological, and genetic) supports the hypothesis of shared pathophysiological underpinnings, no conclusive evidence has yet established a direct or unidirectional causal relationship [22, 90]. Rather, the overlap likely reflects a combination of shared vulnerabilities, mediated through systemic inflammation, gut-brain axis dysfunction, microbial dysbiosis, and neurochemical imbalances (Fig. 1). Whether these converging mechanisms represent true comorbidity, parallel disease expressions, or broader systemic susceptibilities remains to be definitively determined.

#### Sources of Inconsistency and Study Limitations

A major limitation of current research lies in the tendency to conceptualize BD as a homogeneous entity, despite its clinical heterogeneity. No studies to date have systematically examined whether the relationship with IBD differs across BD type I, type II, or cyclothymia. This is a critical gap, as these subtypes exhibit important differences in clinical presentation, biological signatures, treatment response, and comorbidity patterns [3]. Subtype-specific studies could reveal distinct immune or microbiome profiles, offering more refined insights into the biological basis of BD–IBD comorbidity.

Similarly, ethnic and geographic homogeneity in most IBD cohorts limits the generalizability of current findings. Many large-scale genetic and microbiome studies have been conducted in populations of European, East Asian, or North American origin, potentially overlooking population-specific risk variants or protective factors. Given the impact of both genetics and environment on immune function and microbiota composition, future research must prioritize diverse, multi-ethnic cohorts to enhance external validity and uncover context-dependent interactions between BD and IBD. We must stress that several methodological constraints undermine the consistency and interpretability of findings on BD–IBD comorbidity. Most epidemiological investigations have not adequately adjusted for medication effects: mood stabilizers such as lithium and anticonvulsants can alter gut permeability and microbiota composition, while IBD therapies (corticosteroids, immunosuppressants, and biologics) modulate central and peripheral cytokine levels, potentially confounding observed associations with disease activity or inflammatory markers [43, 44, 45, 46]. Lifestyle factors, including smoking status, dietary patterns (e.g., fiber intake, pro-inflammatory fats), physical activity, and substance use, are infrequently measured or controlled for, despite their known influence on both mood regulation and gut inflammation. The presence of comorbid conditions such as metabolic syndrome, obesity, and cardiovascular disease is often overlooked, even though these disorders carry their own inflammatory signatures and may drive spurious correlations with cytokine levels or clinical outcomes [7, 12, 17]. Finally, discrepancies in measurements of the same biomarker, such as IL-6, arise from heterogeneous sampling protocols, assay platforms, and timing relative to disease flares or medication dosing [9, 68, 71]. Together, these limitations highlight the need for future studies to employ standardized protocols, comprehensive covariate adjustment, and stratified analyses to disentangle true pathophysiological signals from treatment, lifestyle, and comorbidity–related confounders.

## Conclusions and Future Directions

Mood disorders are not confined to emotional dysregulation alone but often involve a complex interplay of somatic symptoms, neurovegetative disturbances, and inflammatory processes. This multidimensional clinical presentation reflects the interaction between brain, body, and immune system and contributes to functional impairment and diagnostic delays. Clinicians should adopt a transdiagnostic, integrated approach to symptom assessment, recognizing that physical complaints may represent a gateway to early identification and personalized treatment [7, 16, 17].

Future research should explore biological correlates and longitudinal patterns of these symptoms, as well as the effectiveness of multimodal interventions targeting both mood and somatic domains. It will be essential to establish large, longitudinal cohorts of patients with BD, IBD, and their overlap, integrating serial multi-omics profiling, including genomics, transcriptomics, proteomics, metabolomics, and gut microbiome analyses, with detailed clinical phenotyping of mood symptoms and disease activity. Such studies could reveal biomarkers that predict comorbidity onset, flare interactions, and treatment response, and may uncover distinct endophenotypes (such as lithium-responsive versus non-responsive BD or Crohn’s versus UC), whose specific genetic and inflammatory signatures predispose to the alternate disorder [43].

Building on these observational insights, randomized interventional trials of targeted anti-inflammatory agents (for example, IL-6 or TNF-α inhibitors) in BD populations, and conversely, adjunctive mood-stabilizing therapies like lithium in IBD cohorts, should be designed with rigorous psychiatric and gastrointestinal endpoints [43, 46]. Parallel mechanistic work employing advanced neuroimaging to track neuroinflammation alongside gut-on-a-chip or organoid models will help elucidate the bidirectional gut–brain signaling pathways at play.

Finally, translating these discoveries into practice will require integrated, multidisciplinary care models, combining psychiatry, gastroenterology, nutrition, and telemedicine, to enable early detection, personalized treatment, and continuous monitoring, all of which hold promises for reducing the dual burden of BD–IBD comorbidity and improving patient outcomes.

## Availability of Data and Materials

Not applicable.
